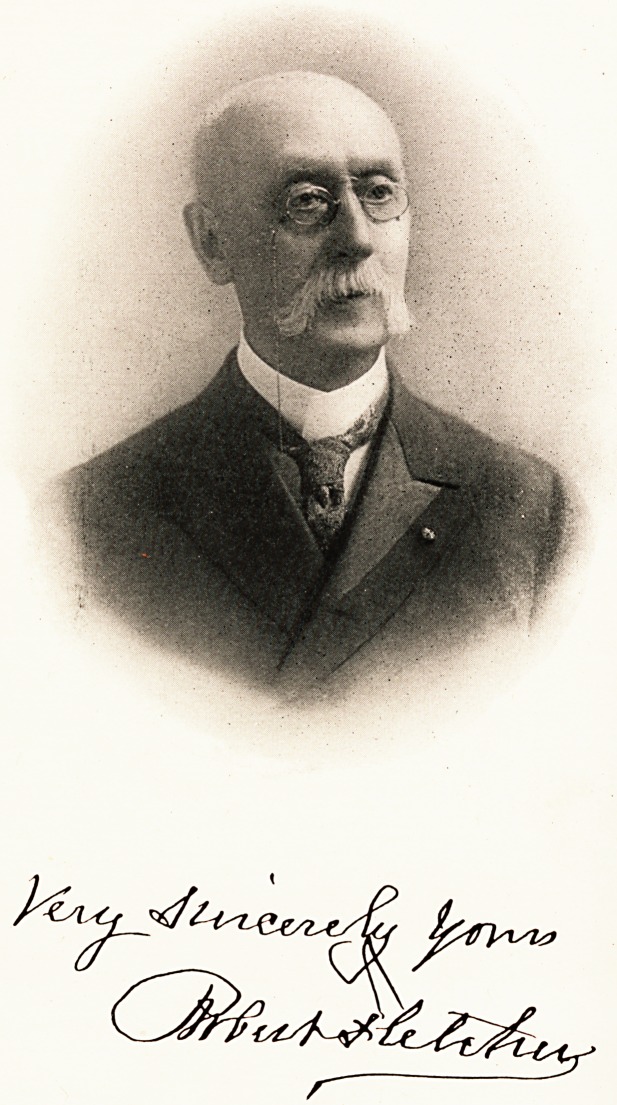# Robert Fletcher

**Published:** 1912-12

**Authors:** William Osler


*Robert Fletcher*

### The Bristol Medico-Chirurgical Journal.

*" Scire est nescire, nisi id me
Scire alius sciret."*

DECEMBER, 1912.

### Robert Fletcher.

In 1881 I paid my first visit to the great Library of the Surgeon-General's Office, Washington, to look up the literature of Echinococcus disease in America, a subject in which I had become interested. At that date the Library had not yet moved from the old Pension Office, and the books had far outgrown the capacity of the building. It was my first introduction to Dr. John S. Billings, at present the head of the Public Library, New York, to whose energy and perseverance the profession of the United States is indebted for one of the greatest collections of medical works in the world. He handed me over to the care of an elderly gentleman, who very quickly put at my disposal the resources of the library, and for two days did everything in his power to further my wishes. This was the beginning of a warm friendship with Dr. Robert Fletcher, and during the thirty years which have since passed I always found him a kindly, wise, and generous adviser in all matters relating to medical bibliography. Probably few men in the profession owe a deeper debt of gratitude to the Surgeon-General's Library than I.

Not only did I enjoy the friendship of the officials of all grades, but from the library itself, and from its two great publications—the *Index Catalogue* and the *Index Medicus*—I have had constant help in my literary work. Among the many congratulations I received last year on the occasion of my baronetcy, none touched me more than a round-robin sent from all the staff of the museum and the library.

The general facts of Dr. Fletcher's life may be briefly told. He was born in Bristol, March 6th, 1823, the son of Robert Fletcher, an accountant, and the business which he founded still continues under the name of Curtis, Jenkins and Co. For a couple of years he was in his father's office, but in 1839 he entered the Bristol Medical School. On June 22nd, 1912, he wrote: "I have always treasured my recollections of the Bristol Medical School and Infirmary with much pleasure and interest. I was articled to Surgeon Henry Clark for the usual five years, the last two of which I spent at the London Hospital, being a pupil of Surgeon James Luke, and of a physician on the medical side whose name I have forgotten."

In 1844 he became a Member of the Royal College of Surgeons and a Licentiate of the Society of Apothecaries. In 1843 he married Hannah, daughter of John Howe, Esq., of Bristol, and in 1847 went to the United States and settled in Cincinnati, where he practised medicine for some years. At the outbreak of the Civil War he was appointed to the first regiment of Ohio Volunteers, and after three years was commissioned Surgeon to the United States Volunteers, and placed in charge of Hospital No. 7, Nashville, Tenn. Subsequently he became Medical Purveyor to the Army at that post. At the end of the war he was breveted Lieutenant-Colonel, and afterwards Colonel, for faithful and meritorious service.

In 1871 he was ordered to Washington to the Provost-Marshall's Office in the War Department, and took part in the preparation of the two volumes of *Anthropometric Statistics* issued by that office in 1875, and contributed to them the special section on Anthropometry.

In 1876 he was transferred to the Surgeon-General's Library.

with which, for the remainder of his long life, he was intimately associated. A collection of books in connection with the Army Medical Department was begun during the Civil War by Surgeon-General Hammond, and continued by his successors, who found it necessary, after the war, to provide literature for the men who were preparing the great work on its medical and surgical history. The collections accumulated, and were housed at first in the Pension Bureau, under the care of Dr. John Billings, who began in 1873 the preparation of the *Catalogue*. Of this work, the famous *Index Catalogue of the Surgeon-General's Library*, Dr. Fletcher was until his death one of the principal editors.

In the preface to the first volume, 1880, Dr. Billings writes: "I wish to specially acknowledge the valuable assistance which I have received from Dr. Robert Fletcher in carrying this volume through the press, assistance which has gone far beyond mere routine or the limits of office hours, and without which I should have found it impossible to have done the work, and to have performed my other official duties."

The work is one of the greatest ever undertaken in the history of bibliography. In the first series of sixteen volumes, completed in 1895, there were indexed of author titles 85,663 volumes and 151,504 pamphlets, and of subject titles 168,557 books and 511,112 journal articles. Of the second series sixteen volumes have already been issued, nearly finishing the letter "S." The thirty-two volumes so far published furnish a medical reference catalogue of colossal proportions—of 286,255 book titles and 1,006,355 journal articles. Each volume contains nearly a thousand pages, closely printed, with from fifty to seventy-five separate references on each.

Those of us who have made frequent use of the *Index Catalogue* much have been impressed with one point—its extraordinary accuracy. In my experience it has been very rare indeed to find a mistake in the reference, either to the name of the author or to a journal. For this admirable accuracy we are indebted to the extraordinary care with which Dr. Billings, Dr. Fletcher, and Dr. Garrison have read the proofs.

The Army practice has been to assign a special surgeon to take charge of the library, over which he exercises general supervision. Since 1904 Colonel McCaw has been in charge, and his kindly interest and care of Dr. Fletcher have been much appreciated by all his old friends.

In 1879 Dr. Billings began the publication of the *Index Medicus,* an index month by month of the current medical literature of all countries. With this Dr. Fletcher was associated as co-editor for twenty-one years, and as editor-in-chief for nine years, having the active help of his friend and colleague Dr. Fielding H. Garrison. Of this work we may repeat what Dr. Billings said of the *Index Catalogue:* "The accuracy and typographical excellence of the volumes are largely due to Dr. Fletcher's careful and skilful supervision."

Perhaps from his early associations with the Law, Dr. Fletcher always took a deep interest in Medical Jurisprudence, on which subject he lectured for some years at the Columbian University, Washington. After the organisation of the Johns Hopkins Hospital Medical School he was appointed Lecturer on Jurisprudence there, and held the position from 1897 till 1903. He was a clear and attractive lecturer, and very popular with the members of his class.

Outside of his bibliographical work, Dr. Fletcher was specially interested in the subject of Anthropology, and contributed largely, as already mentioned, to the two volumes of *Anthropometrical Statistics* issued from the War Department in 1875. In 1882 he wrote a monograph on "Prehistoric Trephining and Cranial Amulets"; in 1883, "Human Proportion in Art and Anthropometry," and "Tattooing among Civilised Peoples"; in 1889, "Myths of the Robin Redbreast in Early English Poetry"; in 1891, "The New School of Criminal Anthropology"; in 1895, "Anatomy in Art"; in 1897, "Scopelism," the first paper on the subject in English. A paper on "Columns of Infamy" will appear in the *American Anthropologist,* and his essay on "Some Diseases Bearing the Names of Saints" appears in this issue of the *Journal.*

From its start Dr. Fletcher took a great interest in the

Johns Hopkins Historical Society, to which he contributed a number of valuable papers, of which the best were his essays on "Medical Lore in the Older English Dramatists and Poets" (1895), "The Witches' Pharmacopœia" (1896), and "A Tragedy of the Great Plague at Milan in 1630" (1898).

Dr. Fletcher's was an interesting and striking personality. Above the average height, always "well groomed," and with a dignified military bearing, age made him a typical courtly gentleman of the old school. He had a rare gift for friendship, and not only his associates in the library, but many of his colleagues in Washington, particularly Dr. Yarrow, men interested in libraries as the late Jas. R. Chadwick, of Boston, and all of his colleagues at the Johns Hopkins Hospital were devoted to him. After his Jurisprudence lecture at the Johns Hopkins Hospital, at the hospitable board of the Director, Dr. Hurd, many of us would gather, delighted to hear Dr. Fletcher's reminiscences of the profession, which went back to the forties. He had met Sir Astley Cooper, and he knew well the famous old men of the Bristol School, and could tell tales of the Middle West in the palmy days of Drake and Dudley and Caldwell. It was a rare treat to dine with him quietly at his club in Washington. He knew his Brillat-Savarin well, and could order a dinner that would have made the mouth of Cœlius Apicius to water.

All the honours came that should accompany old age. In 1906 he was given a banquet by the leading members of the profession in the United States, in recognition of his work at the Surgeon-General's Library. In 1910 the Royal College of Surgeons of England awarded him the honorary medal, which is given at irregular intervals to those who have made "labours, researches and discoveries eminently conducive to the improvement of natural knowledge and of the healing art."

No man ever aged more gracefully. He stuck to his work to the last. In the spring of 1911 he had a bad attack of diphtheria, from which he recovered very slowly, but he insisted on returning to the library, and read proofs to within a few days of his death. Not long before the end his friend and colleague, Dr. Garrison, wrote : "Even on his grey days his wonderful will power and stoicism are something to command admiration. You have probably heard his favourite therapeutic *argumentum ad baculinum* for any bodily ailment, 'Treat it with contempt.'"

His name will be permanently associated in medical literature with the Surgeon-General's Library, and with the great works to which he was so important a contributor.

William Osler.

---

Note from an appreciation by Fielding H. Garrison, M.D., Washington, D.C. :

"With the death of Dr. Robert Fletcher, Principal Assistant Librarian of the Surgeon-General's Library for nearly thirty-five years, there passed away a Nestor of medicine who was looked up to and honoured everywhere for his intensive labours in medical bibliography, his rare scholarship and that courteous and cheerful spirit of helpfulness which, in the words of Dr. Howard Kelly, 'has endeared him to the entire profession of the United States.' Almost ninety when he died, his span of life, like Heberden's, extended over nearly a century, and with Heberden the scholar, as distinguished from the clinician, he had many points of resemblance. In his lifetime he had seen and met personalities so various and so widely separated in time as Sir Astley Cooper and Halsted, Daniel Drake and Sir William Osler, Emerson and the philosophers and poets of a later period. A native of England, he had served his adopted country for fifteen years with distinction as an army surgeon during the Civil War and afterward, and he was buried at Arlington with the honours commensurate with the military rank he attained."—*Journal of the American Medical Association*, November 23rd, 1912.

## Figures and Tables

**Figure f1:**